# Enhancing Essential Oil Extraction from Lavandin Grosso Flowers via Plasma Treatment

**DOI:** 10.3390/ijms25042383

**Published:** 2024-02-17

**Authors:** Ricardo Molina, Carmen López-Santos, Karina Balestrasse, Ana Gómez-Ramírez, Jordi Sauló

**Affiliations:** 1 Department of Biological Chemistry, Institute of Advanced Chemistry of Catalonia (IQAC), Spanish National Research Council (CSIC), 08034 Barcelona, Spain; 2 Nanotechnology on Surfaces and Plasma Group, Institute of Materials Science of Seville (US-CSIC), 41092 Sevilla, Spain; mclopez@icmse.csic.es (C.L.-S.); anamgr@us.es (A.G.-R.); 3 Departamento de Física Aplicada I, Escuela Politécnica Superior, Universidad de Sevilla, 41011 Sevilla, Spain; 4 Instituto de Investigaciones en Biociencias Agrícolas y Ambientales (INBA), Facultad de Agronomía, Universidad de Buenos Aires (UBA), Consejo Nacional de Investigaciones Científicas y Técnicas (CONICET), Buenos Aires C1417DSE, Argentina; kbale@agro.uba.ar; 5 Cátedra de Bioquímica, Departamento de Biología Aplicada y Alimentos, Facultad de Agronomía, Universidad de Buenos Aires, Buenos Aires C1417DSE, Argentina; 6 Departamento de Física Atómica, Molecular y Nuclear, Facultad de Física, Universidad de Sevilla, 41012 Sevilla, Spain; 7 Laboratory of Dioxins, Department of Environmental Chemistry, Institute of Environmental Assessment and Water Research (IDÆA), Spanish National Research Council (CSIC), 08034 Barcelona, Spain; jordi.saulo@idaea.csic.es

**Keywords:** essential oil, Lavandin Grosso, hydrodistillation, plasma technology, surface modification, GC/MS, ATR-FTIR, XPS, SEM, contact angle

## Abstract

This study explores the impact of plasma treatment on Lavandin Grosso flowers and its influence on the extraction of essential oils (EOs) via hydrodistillation. Short plasma treatment times enhance the yield of EO extraction from 3.19% in untreated samples to 3.44%, corresponding to 1 min of plasma treatment, while longer treatment times (10 min) show diminishing returns to 3.07% of yield extraction. Chemical characterization (GC/MS and ATR-FTIR) indicates that plasma treatments do not significantly alter the chemical composition of the extracted EOs, preserving their aromatic qualities. Investigations into plasma–surface interactions reveal changes at the nanometer level, with XPS confirming alterations in the surface chemistry of Lavandin Grosso flowers by reducing surface carbon and increasing oxygen content, ultimately resulting in an increased presence of hydrophilic groups. The presence of hydrophilic groups enhances the interaction between the surface membrane of the glandular trichomes on Lavandin Grosso flowers and water vapor, consequently increasing the extraction of EOs. Furthermore, microscopic SEM examinations demonstrate that plasma treatments do not affect the morphology of glandular trichomes, emphasizing that surface modifications primarily occur at the nanoscale. This study underscores the potential of plasma technology as a tool to enhance EO yields from botanical sources while maintaining their chemical integrity.

## 1. Introduction

*Lavandula* (common name lavender) is a genus of around 30 known species and subspecies and numerous hybrids of flowering plants and belongs to the *Lamiaceae* plant family [1,2]. *Lavandula* species are among the most useful aromatic and medicinal plants with great economic value in perfumery, aromatherapy, cosmetics, pharmaceutical, and food industry, mainly due to the commercial significance of lavender’s essential oil [2,3]. The most common species in cultivation are *Lavandula angustifolia*, *Lavandula x intermedia* (lavandin, hybrid), and *Lavandula latifolia*. Among the main lavandin varieties (Grosso, Abrial, Super), the Grosso variety is the most famous for its essential oil yield and its odor [4]. Additionally, Lavandin Grosso essential oils have phytotoxic properties [5], antiplatelet/antithrombotic properties [6], antimicrobial activity [7,8], and antioxidant [9] and antifungal properties [10,11], to name a few. Lavender essential oil is located in a round shape of glandular trichomes with an average diameter of 100 μm present on the outer surface of the flower [12]. Generally, the essential oil (EO) is isolated from lavender mostly by hydrodistillation or steam distillation of fresh or dried flowers. During the distillation process, the essential oil is heated and flows out through the cuticle of the glandular trichomes to the vapor phase [12,13].

Plasma, known as the fourth state of matter, is naturally present in the universe (e.g., aurora borealis) but can be generated artificially by an electrical discharge in a gas (e.g., neon tubes). Plasma is composed of different types of reactive species (e.g., radicals, electrons, ions, and atoms) and UV–visible radiation. The modifications produced by these chemical species, such as oxidation and the generation of hydrophilic chemical groups, are limited to a few nanometers from the surface of a material in contact with a plasma and therefore do not alter the internal properties of the material. Low-temperature or non-thermal plasmas (NPTs) are widely used for polymeric material treatments, biomaterials, sterilization, or medicine [14,15]. The application of NTPs in agriculture is a recent and rapidly growing field [16,17]. NTP technology is environmentally friendly and can be economically competitive compared to traditional chemical treatments. The application of plasma treatments in the modification of raw materials for essential oil extraction has emerged as an intriguing and promising possibility. Recent studies have successfully employed plasma technology to improve the extraction yield of essential oils from plants, including lemon peel and leaves [18,19,20], grape and tomato pomace [21,22], *Camelina sativa* and cumin seeds [23,24], fennel seeds and spearmint leaves [25], betel leaves [26], and thyme leaves [27]. In the field of extracting natural compounds, including essential oils, the application of plasma treatments prior to the extraction process has shown the potential to alter the chemical and physical properties of plant raw materials, which could influence the efficiency and quality of the subsequent extraction process [16,17]. In particular, microwave-excited low-pressure plasma treatments, as well as atmospheric pressure dielectric barrier discharge (DBD) plasma treatments, have mainly induced small defects such as cracks in the epidermal cell structure along with a drying of the peel directly related to an improved oil extraction process [19,26]. In addition, the formation of hydrophilic groups on the surface has also been related to improved essential oil extraction yield [22,27].

The present study focuses on the application of low-temperature and pressure plasma treatments to Lavandin Grosso flowers as a precursor to enhance the extraction of essential oils. Via a systematic approach, possible changes in the chemical profiles of essential oils will be investigated, along with the physico-chemical changes induced by low-temperature and pressure plasma treatment on the surface of Lavandin Grosso flowers. Ultimately, this study aims to shed light on the potential of plasma technology as a promising tool to improve the extraction of essential oils from plant materials while providing valuable information on how plasma treatment parameters can influence the resulting chemical and aromatic profiles.

## 2. Results and Discussion

### 2.1. Hydrodistillation Kinetics and Characterization of Essential Oils from Lavender Flowers

**Figure 1:**
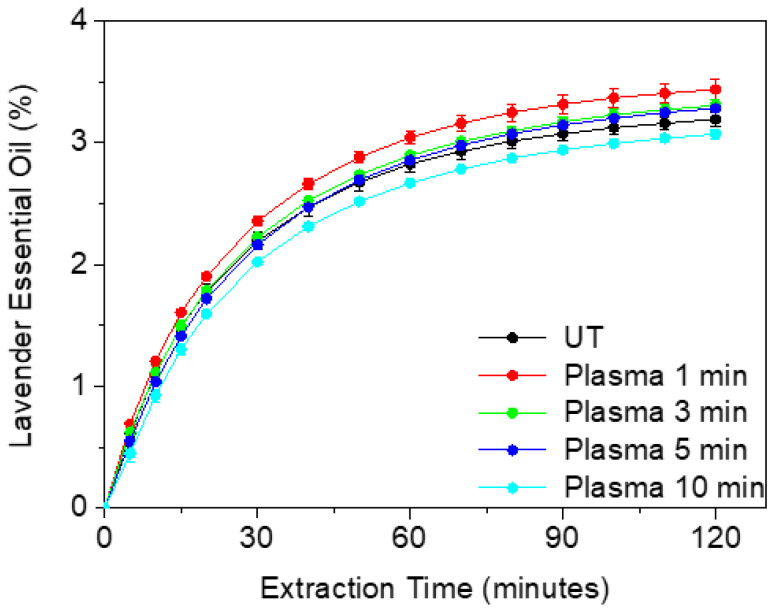
Kinetics of essential oil extracted by hydrodistillation from untreated (UT) and plasma-treated Lavandin Grosso flowers for increasing treatment times.

Hydrodistillation extraction kinetics of the cumulative essential oil (EO) compounds of untreated and plasma-treated Lavandin Grosso flowers is shown in Figure 1. Untreated Lavandin Grosso extraction yield (final percentage of essential oil extraction weight respect total flower weight) after 2 h (3.19 ± 0.06%) agrees with previously reported values in Lavandin Grosso flowers (3.2%) [28]. It can be observed that the initial rate (curve slope in the first 20 min) and the oil extraction yield after 2 h increase with 1 min of plasma treatment (3.44 ± 0.08%). A subsequent increase in plasma treatment time tends to decrease both the initial rate and oil extraction yield with values of 3.31 ± 0.05% and 3.28 ± 0.02% for 3 min and 5 min, respectively. At 10 min plasma treatment time, the rate and the extraction yield obtained (3.07 ± 0.04%) are lower than the corresponding for untreated flowers. Therefore, while short plasma treatment times are beneficial for increasing the amount of essential oils extracted by hydrodistillation, long plasma treatment times (10 min) have a detrimental effect on extraction.

**Figure 2:**
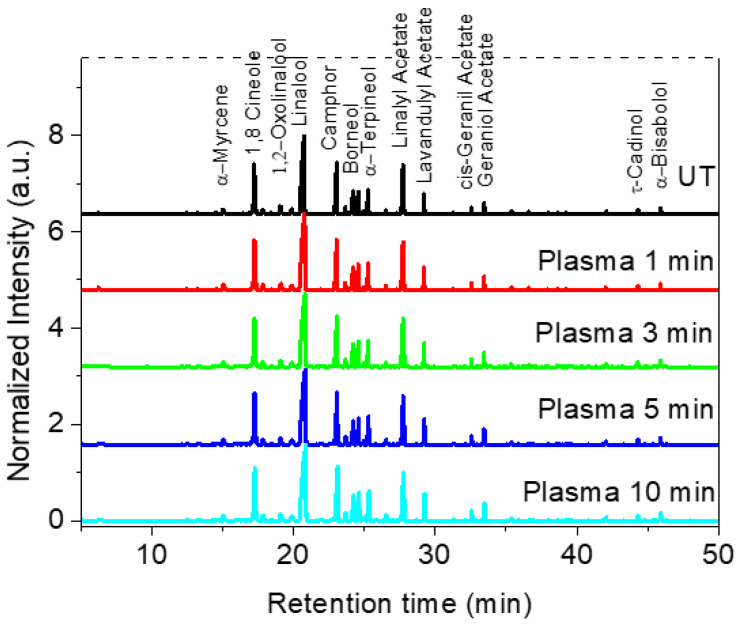
GC/MS chromatogram for essential oils extracted from untreated (UT) and plasma-treated Lavandin Grosso flowers for different treatment times.

The identification of essential oils composition was carried out to determine if plasma active species can chemically modify the oils present inside the glandular trichomes of Lavandin Grosso flowers and therefore alter their fragrance. Figure 2 shows the GC/MS analysis performed on the cumulative essential oils obtained after hydrodistillation (2 h) of untreated and plasma-treated Lavandin Grosso flowers. 1,8-Cineole (Eucalyptol), linalool, camphor, and linalyl acetate are the most intense compounds observed in the gas chromatogram. No significant difference in the intensity of the peaks is observed between oils extracted from untreated or plasma-treated Lavandin Grosso flowers, even for long treatment times (10 min).

Detailed identification of the chemical compounds (Table S1) has evidenced that Lavandin Grosso EOs are composed of ~100 chemical compounds. The chemical composition of Lavandin Grosso EOs depends on the lavender species (*Lavandula angustifolia*, *Lavandula latifolia,* and *Lavandula hybrid* are the most employed in the perfume and cosmetics industry), the geographic region of origin, altitude, soil, and climate conditions, morphology, processing of the plant material, its nature (fresh or dried), and extraction method [3,29,30,31,32,33,34,35,36]. Although with different concentrations, which are attributed to the different parameters described above, the most representative chemical compounds found in the EOs extracted from untreated Lavandin Grosso flowers (Table 1) are in concordance with that of EOs found by other authors [3,4,37,38]. It is important to remark that no new chemical compounds appear, there is no significant variation in chemical composition concentrations, and no new chemical compounds are formed as a result of possible oxidative or reductive chemical reactions of the plasma active species with the EOs located inside the glandular trichomes. The same effect has been previously reported in essential oils extracted from plasma-treated fennel seeds and plasmas-treated spearmint leaves where no significant differences in extracted EO chemical composition are observed between untreated and plasma-treated samples [25].

These results seem to evidence that in the experimental conditions used in this work, at short plasma treatment times (<3 min), active species just modify the surface membrane of the glandular trichomes, modifying their permeability and favoring the diffusion of EOs towards the exterior of the glandular trichomes, thereby increasing the EO extraction yield in this way.

A detailed statistical analysis of the results presented in Table 1 shows a high correlation of the areas corresponding to the different EOs between the untreated and plasma-treated samples (Table 2), indicating that statistically, the plasma treatments scarcely modify the chemical composition of the extractedEOs.

Additionally, it was analyzed by means of linear regression fitting whether individually the area of the different EOs extracted in each sample showed any trend as a function of the plasma treatment time. Table 3 shows the values corresponding to the Pearson coefficients and the adjusted R-squared of the different samples analyzed. A significant trend (r > ±0.70) can be seen in the decrease in the concentrations of Linalyl acetate, 1,8-Cineole, and Limonene as a function of treatment time (negative values of r) and a tendency to increase the area as a function of treatment time forEOs, Terpinen-4-ol, Lavandulyl acetate, Lavandulol, and α-Terpineol (positive values of r). However, taking into account the adjusted R-squared values, it can be observed that only α-Terpineol presents a statistically significant value (0.95838) and that the variation in the concentration of this compound represents only a variation of 0.46% of the total (4.23 for UT sample to 4.69 for 10 min plasma treatment). This slight increase is not considered a significant change from the perspective of applications such as cosmetics, given the wide dispersion of concentrations already observed among essential oils obtained from Lavandin Grosso in different geographical areas [37].

**Figure 3:**
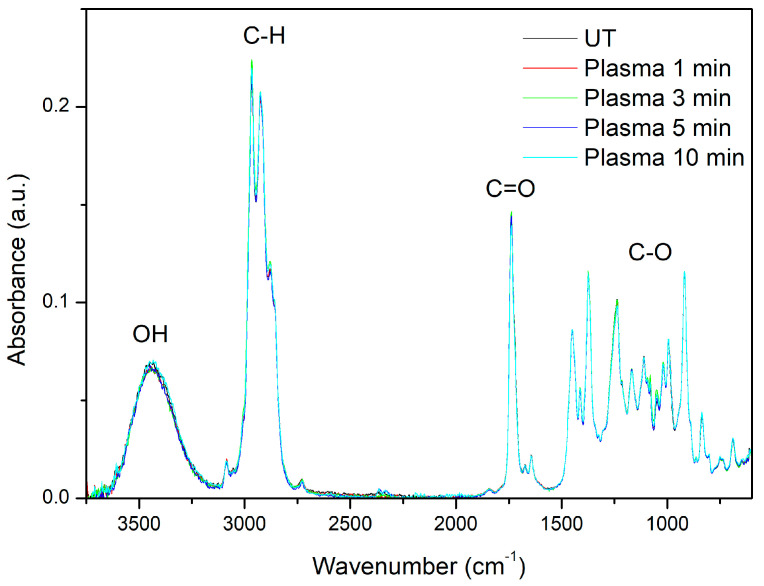
ATR-FTIR spectra of the cumulative essential oils extracted by hydrodistillation of untreated (UT) and plasma-treated Lavandin Grosso flowers for different treatment times.

As shown by GC/MS Lavandin Grosso, EOs are concentrated solutions of volatile compounds with ~100 constituents. Thus, their FTIR spectra are complex due to the spectra of individual components overlapping and the mixing of various vibrational modes [34,39]. However, ATR-FTIR spectra obtained from the essential oil samples display characteristic spectral fingerprints that can be used to discriminate different plant species and chemotypes [40,41,42,43,44,45,46]. ATR-FTIR spectra of the cumulative essential oils extracted by hydrodistillation of untreated and plasma-treated Lavandin Grosso flowers (Figure 3) show characteristic bands corresponding to O–H stretching vibration (3443 cm^−1^), C–H stretching of vinyl groups CH2=CH– typical for the monoterpenes linalool and linalyl acetate (small band at 3085 cm^−1^), symmetric and antisymmetric C–H stretching of –CH_2_ and –CH_3_ groups of alkane chains (2970–2860 cm^−1^), C=O stretching of ester group corresponding to camphor and lynalyl acetate (1737 cm^−1^), methylene C–H bend (1450 cm^−1^), C–H in-plane bend of vinyl groups (1412 cm^−1^), C–O stretching (1240–1027 cm^−1^), and C–H out-of-plane bend of vinyl groups (995 cm^−1^ and 918 cm^−1^) [4,34,39,41,43,44].

ATR-FTIR spectra of the cumulative essential oils extracted by the hydrodistillation of Lavandin Grosso flowers treated with plasma show a remarkable overlapping of all the peaks with respect to the untreated flowers, confirming that no significant variation in chemical composition can be observed after hydrodistillation. Therefore, the results seem to confirm that plasma active species just modify the surface of the glandular trichomes and do not interact significantly with the essential oils located inside the glandular trichomes.

### 2.2. Characterization of Plasma Active Species Present in the Plasma

**Figure 4:**
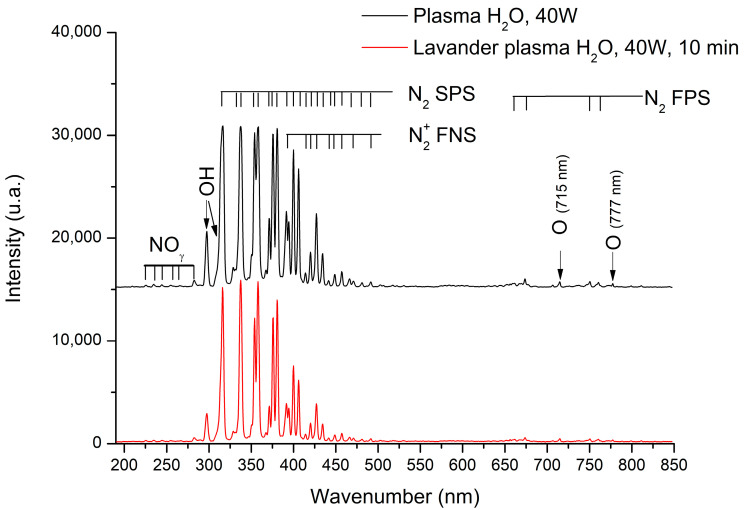
Optical emission spectra of the plasma generated with (red curve) and without (black curve) Lavandin Grosso flowers inside the reactor.

OES (Optical Emission Epectroscopy) is a very powerful method to identify active chemical species present in plasma. The identification of such species can provide information on the plasma/surface reactions that are taking place. The UV-vis emission spectra obtained during the plasma treatment with and without Lavandin Grosso flowers inside the plasma reactor are shown in Figure 4. The emission spectra recorded during the plasma treatment without Lavandin Grosso flowers inside the plasma reactor show different characteristic emission bands associated with nitroxide molecules (NO_ϒ_) in the region 225–283 nm, OH radicals (OH•: 308.9 nm, 287 nm) [47,48], nitrogen molecules (second positive system, N_2_ SPS) in the region 297–430 nm, nitrogen first negative system (N^+^_2_ FNS) in the region 390–490 nm and nitrogen first positive system (N_2_ FPS) in the region 660–760 nm [49,50,51,52], and atomic oxygen lines at 715 and 777 nm [53,54,55,56,57,58]. The presence of OH radicals can be attributed to the water-rich atmosphere employed, whereas the remaining peaks associated with nitrogen and oxygen reactive species result from the presence of air in the reactor environment. The spectrum collected at 10 min of plasma treatment with Lavandin Grosso flowers inside the reactor shows a slight decrease in the peak’s intensity attributed to the smaller effective area of the electrode due to the presence of the Lavandin Grosso flowers on it. However, no emission bands associated with carbon species (CN, CH, and C_2_) are observed [57,59,60], suggesting that EO exudation from the glandular trichomes during plasma treatment is not appreciable in the experimental conditions used in this work, even for the longest time of plasma treatment (10 min).

### 2.3. Weight Loss Tracking of Lavandin Grosso Flowers Treated with Plasma

**Figure 5:**
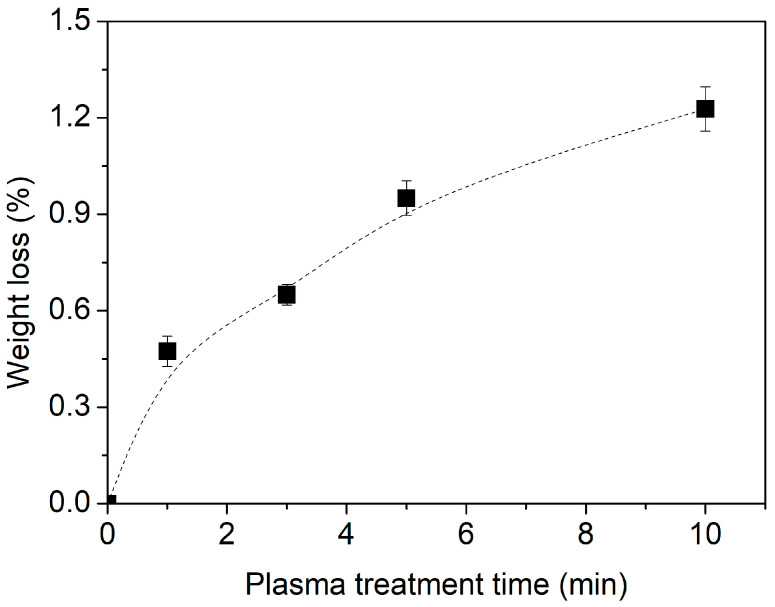
Lavandin Grosso flowers’ weight loss (%) as a function of plasma treatment time.

Biopolymeric materials exposed to plasmas or low-pressure conditions usually exhibit a measurable weight loss due to different physical or chemical processes such as water desorption and/or hydrocarbon oxidative etching [61,62,63]. Figure 5 depicts the weight loss for Lavandin Grosso flowers subjected to the different plasma treatment times used in this work.

As observed, Lavandin Grosso flowers experience progressive weight loss as the treatment time increases, reaching a maximum of 1.2% at 10 min. It is worth mentioning that despite a minor weight loss (approximately 0.5%) found for 1 min treatment, the essential oil yield values depicted in Figure 1 for this treatment (3.44%) exceed those of the untreated sample (3.19%). This suggests that the weight loss primarily stems from the removal of water content within the flowers. On the other hand, the slight increase in weight loss (Figure 5) and the progressive decrease in the extraction yield of the essential oils (Figure 1) with treatment time could indicate that in addition to the water loss process, there could be other processes such as exudation of the essential oils during plasma treatment or possible chemical modification of the essential oils located within the glandular trichome of the Lavandin Grosso flower such that they are not easily evaporated during the hydrodistillation process and therefore cannot be detected in the extracted essential oil.

### 2.4. Wettability and Water Vapor Adsorption of Plasma-Treated Lavandin Grosso Flowers

**Figure 6:**
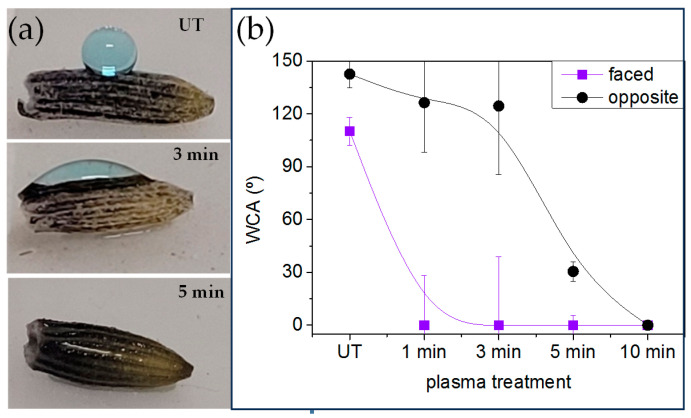
(**a**) Images of small water droplets placed onto plasma-treated Lavandin Grosso flowers and (**b**) evolution of water contact angle with the plasma treatment time for two different water droplet locations (the region in direct contact with the plasma-faced and the region in contact with the electrode (opposite)). Note: Lines representing standard deviations have been intentionally truncated in some data points for visual clarity. Corresponding error bars (upper or lower) are maintained for some data points. The untreated sample is denoted by the abbreviation UT.

To further investigate the mechanism by which Lavandin Grosso essential oil extraction is enhanced by short plasma treatment times, the interaction of Lavandin Grosso flowers with water and water vapor occurring during hydrodistillation was evaluated by means of water contact angle and water vapor adsorption. Small droplets placed onto Lavandin Grosso flowers can be visualized in Figure 6, and the apparent water contact angle value is obtained from these images. Due to the small size of dry Lavandin Grosso flowers (~5 mm long and ~1 mm width) and the high curvature of their surface, the apparent water contact angle is strongly affected by the location where the small water drop is placed onto the flower, and therefore the results obtained can only be interpreted qualitatively. Taking this into account, untreated Lavandin Grosso flowers evidence a hydrophobic apparent water contact angle (126.5°) that can be related to their surface chemical composition and to their high topographic surface roughness [64]. On this basis, plasma treatment progressively affects apparent water contact angle and superhydrophilic behavior can be observed if water droplet is deposited onto a flower area exposed directly to plasma active species. However, as can be observed in Figure 6b, a flower area not directly exposed to plasma active species (i.e., part of the flower leaning onto the lower electrode) preserves a more hydrophobic behavior. Nevertheless, the apparent water contact angle measured on those “hidden” regions tends to decrease as a function of plasma treatment time becoming hydrophilic after 5 min of plasma treatment. Although the apparent water contact angle measured on Lavandin Grosso flowers can just report qualitative results, it is clear that plasma treatment noticeably modifies the wetting behavior from hydrophobic to superhydrophilic. Additionally, the induced surface chemical and/ or topographic heterogeneity (differences between changes in the faced and opposite regions to the plasma discharge) of Lavandin Grosso flowers tend to decrease as a function of plasma treatment time [62,65,66].

**Figure 7:**
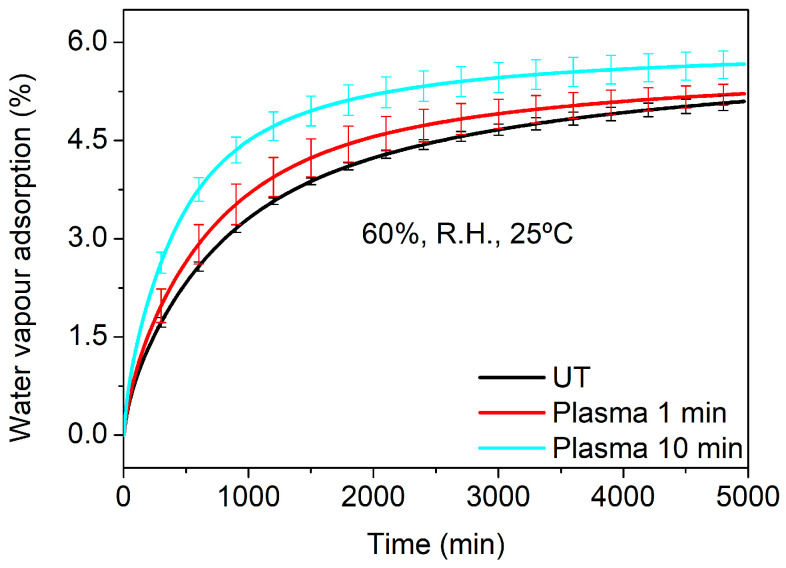
Water vapor adsorption (at 60% R.H. and 25 °C) for untreated (UT) and plasma-treated Lavandin Grosso flowers at short and long treatment times (1 min and 10 min).

During the hydrodistillation process, the surface of Lavandin Grosso flowers also interacts with water vapor. Thus, the water vapor adsorption of untreated and plasma-treated Lavandin Grosso flowers at short and long treatment times (1 and 10 min, respectively) was evaluated. In order to minimize the evaporation process of the essential oil that would overlap with the water adsorption process under the conditions used in the hydrodistillation (100 °C and 100% R.H.), milder humidity and temperature conditions have been chosen during the dynamic vapor sorption (DVS) measurements (25 °C and 60% R.H.). Figure 7 shows that plasma treatment of Lavandin Grosso flowers increases both the water vapor adsorption rate (higher slope in the curve) and the final water vapor yield, evidencing an increase in the interaction of the Lavandin Grosso flower surface with water vapor. This behavior correlates with the increase in hydrophilicity observed by means of the contact angle and suggests that the interaction between water molecules (vapor or liquid) and glandular trichomes is favored, increasing desorption of aromatic oils in a more effective way in Lavandin Grosso flowers treated with plasma.

### 2.5. XPS Surface Chemistry Analysis of Lavandin Grosso Flowers Treated with Plasma

The modification of the surface chemical composition induced on plasma-treated Lavandin Grosso flowers was determined by XPS. Table 4 shows the elemental chemical composition as a function of plasma treatment time. Untreated Lavandin Grosso flowers show a high content of surface carbon (~88%), oxygen (~10%), and traces of other elements (Ca, Si, and Al). Based on the high apparent contact angle (~125 °) of the untreated Lavandin Grosso flowers, it is suggested that the outermost part of the flower surface is mainly composed of hydrocarbon groups (C-C and C-H), which are hydrophobic in nature and combined with the high surface roughness of the Lavandin Grosso flowers give a hydrophobic behavior. Depending on the time of treatment with water vapor plasma, a progressive decrease in the carbon content and an increase in surface oxygen are observed, while the chemical composition associated with the traces (Ca, Si, and Al) remains practically constant.

**Figure 8:**
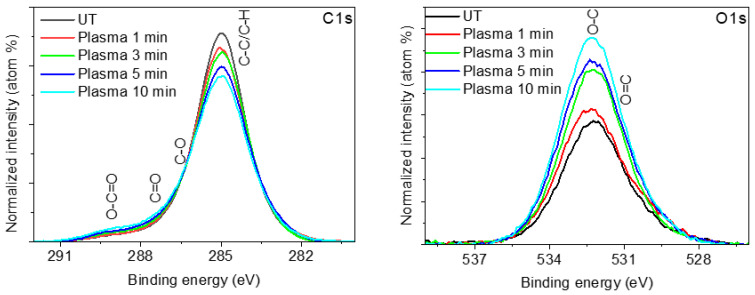
XPS high-resolution C_1s_ and O_1s_ spectra corresponding to untreated (UT) and plasma-treated Lavandin Grosso flowers for different treatment times.

The high-resolution spectra corresponding to the C_1s_ photoelectron peaks (Figure 8) show a progressive decrease in the C-C and C-H groups at 285.0 eV and an increase in hydrophilic surface groups (C-O at 286.4 eV, C=O at 288.3 eV, and O-C=O at 289.4 eV) as a consequence of the progressive oxidation of the C-C and C-H groups induced by active oxygen-containing plasma species (OH and O) [62,63,67,68]. The formation of hydrophilic species such as hydroxyl (-OH) or carboxylic (-O-C=O) ones is more evident in the O_1s_ photoelectron peak where a progressive increase in O=C groups at 531.5 eV and O-C groups at 533.0 eV are observed as a function of plasma treatment time.

### 2.6. Morphological Observation of Plasma-Treated Lavandin Grosso Flowers

**Figure 9:**
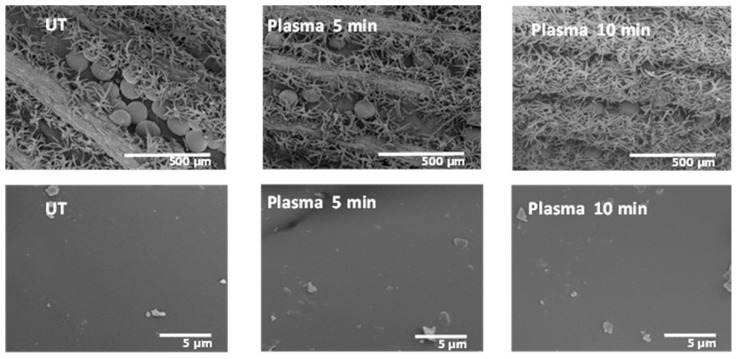
SEM images for 2 different resolved scales corresponding to untreated (UT) and plasma-treated Lavandin Grosso flowers for 5 and 10 min of treatment times.

The morphological changes on the surface of the Lavandin Grosso flowers as a consequence of the plasma treatment were evaluated by SEM. Figure 9 reveals that the untreated Lavandin Grosso flowers show row-like grooves in the interior, of which spherical glandular trichomes appear to be anchored to the surface of flowers by bush-like trichomes. The surface of the glandular trichomes is very smooth, with impurities of biological material on the surface. Plasma treatments do not change the surface roughness of the glandular trichomes even at long treatment times, showing that the surface modifications induced by the plasma active species occur preferentially at the nanometer level.

Unlike what was hypothesized in previous works [18,22,23,24,26], where the enhancement of the essential oil extraction rate is attributed to an aggressive physical modification of the peel surface, Lavandin Grosso glandular trichomes morphology is preserved after the water vapor plasma treatments. However, slight peel surface oxidation induced by the plasma discharge seems to induce better oil diffusion via the extraction process. New oxygenated functionalities anchored to the surface membrane of the glandular trichomes on Lavandin Grosso flowers may facilitate the water permeability pointing to improve the hydrodistillation process [22,24].

## 3. Materials and Methods

### 3.1. Materials and Sample Preparation

Healthy, clean, and dried Lavandin Grosso flowers supplied by Gran Velada S.L. (Magallón, Zaragoza, Spain) were used in this work. Hexane (SupraSolv, Merck, Darmstadt, Germany) was used in essential oil separation and gas chromatography/mass spectrometry (GC/MS) analysis. Methylene blue (Certified by the Biological Stain Commission, Sigma-Aldrich, Burlington, MA, USA) was used as a visual contrast agent in a distilled water solution for measuring water contact angles.

### 3.2. Low-Temperature and Pressure Plasma Treatment

In this work, a dielectric barrier discharge reactor operating in the low-pressure regime (~230 Pa, water vapor pressure at 20 °C) was used. During plasma treatment of polymeric or biological materials, water desorption usually occurs, which contaminates the gas [61,62,63]. For this reason, a mixture of water vapor with the residual air contained in the reaction chamber was chosen as the plasma-generating gas. To obtain a constant atmosphere rich in water vapor between the electrodes, a sealed flask containing distilled water was connected to the reactor chamber, and a vacuum was created until the water vapor pressure was reached. At this point, the water starts to bubble as a result of the degassing of the gases dissolved in the water, predominantly CO_2_. Plasma ignited just after the water stopped bubbling. The reactor consists of two 36 cm diameter circular aluminum electrodes covered with glass, creating a fixed gap of 1 cm for plasma generation. Lavandin Grosso flowers were placed onto the lower electrode covering almost all the electrode area. The plasma discharge was initiated by a 16 kHz sinusoidal signal generated using a GF-855 function generator (Promax, L’Hospitalet de Llobregat, Spain) connected to an AG-1012 linear amplifier (T&C Power Conversion, Inc., Rochester, NY, USA). A matching network and two transformers (HR-Diemen S.A., Sant Hipòlit de Voltregà, Spain) were connected to the output of the amplifier to increase the voltage up to 20 kV. Plasma treatments were carried out at 40 W and different times ranging from 1 min to 10 min.

### 3.3. Hydrodistillation of Lavandin Grosso Flowers

For hydrodistillation kinetics, Lavandin Grosso flowers (100 g) were immersed in 1200 mL of distilled water in a round bottom flask (2000 mL) and heated to 100 °C in a 2000 mL heating mantle (J.P. Selecta, Abrera, Barcelona, Spain). After vapor condensation in a water-refrigerated tube (30 cm), the extracted solution (water and essential oil) was collected in individual vials at different extraction times (steps of 5 min in the interval 5 min to 30 min and steps of 10 min in the interval 30 min to 120 min). Hexane (5 mL), in which the essential oil is soluble, was added to the extracted solution. Separation of the water and organic phases was performed using a 25 mL burette and collected in different glass vials. The essential oil was obtained after complete solvent evaporation (24 h) in the air at room temperature (20 °C) and weighed in a balance. Triplicates for every extraction time were performed. After 2 h of extraction, no significant rise in the extracted essential oil was observed. To obtain the essential oil extracted during the whole process, an additional hydrodistillation was performed, and the content of the organic phase of each vial, after separation of the water and organic phases, was mixed in a single vial (accumulated essential oil) and stored hermetically sealed in a refrigerator (4 °C) until further measurements (GC/MS and ATR-FTIR).

### 3.4. Gas Chromatography/Mass Spectrometry Analysis (GC/MS)

The analysis of the cumulative essential oil was carried out by GC/MS. The mass spectrometer used was a Thermo Scientific ISQ single quadrupole coupled with a TRACE Ultra gas chromatograph. The GC column used was DB5 MS 60 m × 0.25 mm i.d. × 0.25 μm film capillary column (Agilent, Santa Clara, CA, USA), with a splitless inlet mode at 1 mL/min of flow rate with helium as carrier gas, with constant flow. The injector temperatures were 280 °C. The oven temperature was programmed from 50 °C (3 min) to 230 °C at a rate of 3 °C /min, and then from 230 to 310 °C at a rate of 30 °C /min during 8 min. The injection volumes of essential oil diluted in hexane (1:10) were 1 µL. The single quadrupole mass spectrometer was performed with electron ionization (EI) at 70 eV of electron energy, operating in the full-scan acquisition mode in the m/z range of 50–500 and a source temperature of 250 °C. The components of the essential oils were identified by comparison of their mass spectra with those of the spectrometer database using the NIST library. The acquired data Thermo RAW formats (software Xcalibur 3.0.63, Thermo Fisher Scientific, Waltham, MA. USA) were converted to DAT format using the open source software SeeMS ProteoWizard 3.0 (https://proteowizard.sourceforge.io, accessed on 10 January 2024) [69]. The percentages of the EO components were determined using the method of area normalization and without the application of response factor corrections according to standard methods [4,70].

### 3.5. Attenuated Total Reflectance Fourier Transform Infrared Spectroscopy (ATR-FTIR)

ATR-FTIR analysis of the cumulative essential oils extracted by hydrodistillation was carried out using a Nicolet AVATAR 360 spectrometer in the range of 400–4000 cm^−1^. Measurements were performed using the Smart iTR sampling Accessory (Thermo Scientific Inc., USA). A few drops of the accumulated essential oils were deposited on the diamond glass, and FTIR-ATR measurements were performed after hexane evaporation. Spectra were obtained from an average of 32 scans using a resolution of 4 cm^−1^. An advanced ATR correction algorithm (OMNIC 7.3 from Thermo Electron Corporation, Waltham, MA, USA) was used to correct the band intensity distortion, peak shifts, and polarization effects.

### 3.6. Optical Emission Spectroscopy (OES)

OES analysis was used to characterize the plasma active species during Lavandin Grosso flower treatment. Light was collected via an optical fiber located in front of a reactor quartz window. The fiber was connected to a Black Comet spectrometer (Stellarnet, Tampa, FL, USA) with concave gratings. Spectra were recorded in the UV–VIS wavelength range (190–850 nm) with an integration time of 10 s.

### 3.7. Weight Loss Analysis

For the determination of the weight loss (Wloss) induced by the plasma treatment, approximately 10 gr of untreated Lavandin Grosso flowers were weighted in a balance (KERN ABJ 120-4NM, KERN & SOHN GmbH, Balingen, Germany) before (W_i_) and just after (W_f_) the plasma treatments. Averaged weight loss was obtained from 3 replicas per plasma treatment according to the following Equation (1):

(1) $$W_{loss}\left(\%\right)=100\times \frac{W_{i}-W_{f}}{W_{i}}$$

### 3.8. Wetting Behavior of Plasma-Treated Lavandin Grosso Flowers

The wetting behavior of Lavandin Grosso flowers was characterized before and after plasma treatments by measuring with a goniometer the static contact angle of a small droplet carefully deposited onto the flower surface (~2 µL of a 0.5 µM water diluted methylene blue solution). Methylene blue solution was used to better visualize the water drop. The surface tension of these methylene blue solutions at the micromolar level (71.0 mN/m) is practically identical to that of deionized water (72.0 mN/m) [71]. Results are presented as an average of 30 different measurements. Contact angle measurements were performed immediately after plasma treatment.

### 3.9. Dynamic Water Vapor Sorption (DVS)

A thermogravimetric balance, Sorption Analyser Q5000SA (TA Instruments, New Castle, DE, USA), with a controlled humidity chamber, was applied to determine the water absorption kinetics of Lavandin Grosso flowers. Experiments were conducted in triplicate on each sample (~30 mg) with a total gas flow of 200 mL/min at 25 °C and a 60% of relative humidity (R.H.) Samples were subjected to an initial drying at 25 °C and 0% R.H. until their mass reached equilibrium (change in mass of less than 0.01% per minute for 18 min).

### 3.10. X-ray Photoelectron Spectroscopy (XPS)

XPS analysis was carried out in a SPECS spectrometer provided with a hemispherical analyzer (DLSEGD-Phoibos-Hsa3500, SPECS Surface Nano Analysis GmbH, Berlin, Germany). Non-monochromatic Al Kα radiation was employed to record the spectra that were obtained in the constant pass energy mode at a value of 50 eV for the general survey and 30 eV for high-resolution spectra. Calibration in binding energy (BE) was taken at the carbon functional C-H and C-C bonding groups, appearing at 285.0 eV in the C1s zone. The surface composition was estimated after a Shirley-type background subtraction from the area of the different photo-emission peaks modified by their corresponding sensitivity factors [72].

### 3.11. Scanning Electron Microscope Analysis (SEM)

The morphology of untreated and plasma-treated flowers was assessed by using a Hitachi S3500N scanning electron microscope (Hitachi High Technologies Co., Ltd., Tokyo, Japan) at an accelerating voltage of 5 kV in the Institute of Marine Sciences of the Spanish Research Council (CSIC) facilities. Before analysis, samples were coated with a layer of gold (thickness ~20 nm), making use of a Quorum Q150RS gold sputter coater.

### 3.12. Statistical Analysis

Statistical analysis of the presented results has been managed according to the replica of experiments indicated at each methodological process (see Section 3.3, Section 3.7, Section 3.8, and Section 3.9, where statistical treatment was applied for hydrodistillation, weight loss, wetting, and dynamic water vapor sorption analysis) via the mean value and its corresponding standard deviation. Pearson correlation analysis, focusing on the areas of the different EOs obtained for untreated and plasma-treated samples, was performed using Unscrambler 11 software (CAMO AS, Jarlevn. 4, Trondheim, Norway). A linear regression analysis of the areas of the different EOs as a function of plasma treatment time was performed using OriginPro 8.5 (Northampton, MA, USA).

## 4. Conclusions

This comprehensive study extensively explored the impact of plasma treatment on Lavandula Lavandin Grosso flowers and its consequential effects on the extraction of essential oils (EOs) via hydrodistillation. A multifaceted approach, encompassing various analytical techniques, shed light on the intricate relationship between plasma exposure and Lavandin Grosso flower properties, as well as the resulting implications for EO extraction. The findings highlighted the critical importance of optimizing plasma treatment duration, with short durations significantly enhancing EO extraction yield while longer durations resulted in diminishing returns. Furthermore, plasma treatment induced a shift in the surface properties of Lavandin Grosso flowers, transitioning them from hydrophobic to superhydrophilic states, ultimately contributing to a more efficient EO extraction process. Crucially, despite these surface modifications, the chemical composition of the extracted EOs remained largely unaffected, preserving their original aromatic qualities. Detailed investigations at the nanoscale level revealed that plasma-induced modifications primarily occurred at the surface, leaving the macroscopic morphology of Lavandin Grosso flowers largely intact. In summary, this study underscores the potential of plasma treatment as a powerful tool for enhancing EO extraction yields from botanical sources while maintaining their chemical integrity. It offers promising advancements for the essential oil industry and natural fragrance production by achieving a reduction in process time and increasing the extraction yield, both factors that could compete economically with the industrial scalability of plasma technology.

## Figures and Tables

**Table 1:** GC/MS determination of main compounds (area %) in EOs extracted from untreated (UT) and plasma-treated Lavandin Grosso flowers, comparative with ISO normative.

Compound	UT	1 min	3 min	5 min	10 min	Lavandin Grosso, French Type [37]
Linalool	35.61	35.93	35.45	35.71	36.10	24.0–37.0
Linalyl acetate	9.61	8.96	9.58	8.75	8.45	25.0–38.0
1,8-Cineole	10.83	10.61	10.14	10.62	10.03	4.0–8.0
Camphor	10.98	10.02	10.27	10.39	10.34	6.0–8.5
Limonene	0.48	0.52	0.49	0.47	0.44	0.5–1.5
Cis-β-Ocimene	0.73	1.07	1.02	1.04	0.98	0.5–1.5
Terpinen-4-ol	3.63	3.78	3.84	3.82	3.95	1.5–5.0
Lavandulyl acetate	2.69	2.82	2.96	2.95	3.05	1.5–3.5
Lavandulol	1.06	1.09	1.13	1.12	1.18	0.2–1.0
α-Terpineol	4.23	4.32	4.34	4.42	4.69	0.3–1.3
Borneol	4.29	4.03	4.16	4.12	4.25	1.5–3.5

**Table 2:** Pearson correlation analysis attending to the areas of the different Eos obtained for untreated (UT) and plasma-treated samples.

	UT	1 min	3 min	5 min	10 min
**UT**	1.00000	0.99919	0.99949	0.99934	0.99847
**1 min**	0.99919	1.00000	0.99963	0.99989	0.99950
**3 min**	0.99949	0.99963	1.00000	0.99952	0.99917
**5 min**	0.99934	0.99989	0.99952	1.00000	0.99967
**10 min**	0.99847	0.99950	0.99917	0.99967	1.00000

**Table 3:** Values corresponding to the linear regression analysis of areas of the different EOs as a function of plasma treatment time.

Compound	Number of Points	Degrees of Freedom	Residual Sum of Squares	Pearson’s Coefficient (r)	Adjusted R-Squared
Linalool	5	3	0.17464	0.5852	0.12328
Linalyl acetate	5	3	0.40244	−0.78546	0.48926
1,8-Cineole	5	3	0.21091	−0.74332	0.40336
Camphor	5	3	0.46929	−0.25306	−0.24795
Limonene	5	3	0.0011	−0.82236	0.56838
Cis-β-Ocimene	5	3	0.06641	0.33987	−0.17932
Terpinen-4-ol	5	3	0.01172	0.88417	0.70902
Lavandulyl acetate	5	3	0.0175	0.8822	0.70437
Lavandulol	5	3	0.0175	0.8822	0.70437
α-Terpineol	5	3	0.00385	0.98427	0.95838
Borneol	5	3	0.0407	0.23124	−0.26204

**Table 4:** Surface atomic chemical composition (%) of untreated (UT) and plasma-treated Lavandin Grosso flowers determined by XPS.

Sample	C (%)	O (%)	Ca (%)	Si (%)	Al (%)
UT	88.1	9.7	0.4	0.8	0.9
1 min	86.1	11.1	0.6	0.9	1.2
3 min	85.0	13.2	0.4	0.6	0.8
5 min	83.5	14.5	0.5	0.7	0.7
10 min	82.2	15.9	0.3	0.4	0.9

## Data Availability

Data will be made available on request.

## Supplementary Materials

The following supporting information can be downloaded at https://www.mdpi.com/article/10.3390/ijms25042383/s1.
