# Supplementary material for: Enhancing Essential Oil Extraction from Lavandin Grosso Flowers via Plasma Treatment
Source: Int J Mol Sci. 2024 Feb 17;25(4):2383. doi: 10.3390/ijms25042383 (PMC10889515; doi:10.3390/ijms25042383)
Supplement: Supplementary file 1 [file ijms-25-02383-s001.zip › ijms-2849503-supplementary.pdf]

SUPPORTING INFORMATION

Table S1. GC/MS determination of chemical compounds (area %) in Essential Oils extracted from untreated (UT) and plasma treated Lavandin Grosso flowers at different treatment times (1- 10 min).

Retention Time (min)	Compound	UT	1 min	3 min	5 min	10 min
11.91	Tryciclene	0.01	0.01	Trace	Trace	Trace
12.05	α-Thujene	0.03	0.04	0.03	0.03	0.03
12.4	alpha-pinene	0.24	0.26	0.22	0.22	0.18
13.19	Camphene	0.25	0.25	0.22	0.23	0.20
14.25	Sabinene (β-Thujene)	0.06	0.07	0.07	0.07	0.06
14.50	beta-pinene	0.45	0.47	0.43	0.45	0.41
15.00	alpha Myrcene	0.98	1.46	1.37	1.39	1.27
15.97	3-Carene	0.10	0.11	0.11	0.11	0.11
16.11	Acetic Acid. Hexyl ester	0.09	0.11	0.10	0.10	0.10
16.38	alpha-terpinen	0.14	0.16	0.16	0.16	0.15
16.77	o-cymene	0.10	0.11	0.10	0.11	0.10
17.01	Limonene	0.48	0.52	0.49	0.47	0.44
17.22	Eucalyptol	10.83	10.61	10.14	10.62	10.03
17.80	cis-beta-ocimene	0.73	1.07	1.02	1.04	0.98
18.42	γ-Terpinene	0.17	0.19	0.18	0.19	0.18
19.06	1.2-Oxolinalool	1.38	1.40	1.43	1.40	1.39
19.76	α- Terpinolen	0.21	0.22	0.21	0.17	0.18
19.85	Linalol oxide	1.08	1.14	1.17	1.15	1.16
20.74	Linalool	35.61	35.93	35.45	35.71	36.10
21.19	Limonen-6-ol. pivalate	0.05	0.06	0.06	0.06	0.05
21.86	1.5.5-Trimethyl-6-methylene-cyclohexene	0.08	0.15	0.14	0.15	0.14
21.97	Cosmene	0.06	0.08	0.07	0.08	0.07
22.53	Pinane	0.04	0.04	0.04	0.04	0.04
22.62	Isopinocarveol	0.10	0.10	0.09	0.09	0.09
22.78	n-Hexyl isobutyrate	0.11	0.12	0.13	0.13	0.13
23.02	Camphor	10.98	10.02	10.27	10.39	10.34
23.32	Ethyl linalool	0.07	0.07	0.07	0.07	0.07
23.64	Lavandulol	1.06	1.09	1.13	1.12	1.18
24.18	Borneol	4.29	4.03	4.16	4.12	4.25
24.55	Terpinen-4-ol	3.63	3.78	3.84	3.82	3.95
24.93	Butanoic acid. hexyl ester	0.42	0.45	0.45	0.45	0.45
25.26	α-Terpineol	4.23	4.32	4.34	4.42	4.69
25.56	Isoborneol	0.02	0.02	0.02	0.02	0.02
25.81	D-Verbenone	0.10	0.10	0.10	0.10	0.11
26.12	Geranyl vinil ether	Trace	0.01	0.01	0.01	0.01
26.51	cis-Geraniol	0.60	0.61	0.63	0.64	0.70
26.80	Isobornyl formate	0.04	0.04	0.04	0.04	0.04

26.99	Butanoic acid. 2-methyl-. hexyl ester	0.05	0.05	0.05	0.06	0.05
27.29	n-Hexyl iso-valerate	0.12	0.13	0.13	0.12	0.12
27.71	Linalyl acetate	9.61	8.96	9.58	8.75	8.45
27.77	trans-geraniol	1.36	1.40	1.35	1.47	1.55
28.67	Dihydrocarveol	0.03	0.04	0.04	0.04	0.04
29.21	Lavandulyl acetate	2.69	2.82	2.96	2.95	3.05
29.35	Bornil acetate	0.08	0.08	0.08	0.08	0.09
29.99	Carvacrol	0.05	0.06	0.05	0.06	0.06
31.30	Isobutyl tiglate	0.14	0.15	0.16	0.15	0.16
31.41	Z.Z-2.5-Pentadecadien-1-ol	0.03	0.03	0.02	0.02	0.02
31.8	2.5-Octadecadiytioic acid. methyl ester	0.01	0.01	0.01	0.01	0.02
31.99	9-Octadecen-12-ynoic acid. methyl ester	0.01	0.01	0.01	0.01	0.01
32.11	α-Terpinyl propionate	0.03	0.04	0.03	0.04	0.04
32.56	cis-Geranyl acetate	0.80	0.88	0.90	0.93	0.99
32.93	8-Hydroxylinalool	0.03	0.03	0.03	0.03	0.03
33.04	(S)-cis-Verbenol	0.01	0.01	0.01	0.01	0.01
33.20	Geranyl propionate	0.02	0.02	0.02	0.02	0.02
33.45	Geraniol acetate	1.49	1.63	1.67	1.73	1.83
33.57	γ-Muurolene	0.03	0.03	0.02	0.02	0.02
33.82	10-Heptadecen-8-ynoic acid. methyl ester. (E)-	0.02	0.02	0.02	0.02	0.02
33.9	trans-α-Bergamotene	0.03	0.03	0.02	0.02	0.02
34.24	10-Heptadecen-8-ynoic acid. methyl ester. (E)-	Trace	0.01	Trace	Trace	Trace
34.44	11.13-Dihydroxy-tetradec-5-ynoic acid. methyl ester	Trace	Trace	Trace	Trace	Trace
35.04	α-Gurjunene	0.04	0.04	0.04	0.04	0.04
35.29	α-Santalene	0.06	0.06	0.06	0.04	0.04
35.40	beta-Caryophyllene	0.36	0.36	0.34	0.27	0.23
35.87	trans-α-Bergamotene	0.04	0.05	0.04	0.03	0.03
36.19	cis-β-Farnesene	0.04	0.05	0.04	0.04	0.03
36.62	trans-β-Farnesene	0.29	0.26	0.24	0.18	0.14
36.82	Geranyl isobutyrate	0.05	0.05	0.05	0.05	0.05
36.92	cis-α-Bisabolene	0.03	0.03	0.03	0.02	0.02
37.18	β-Cuvebene	0.02	0.02	0.02	0.02	0.02
37.74	α-Longipinene	0.01	0.01	0.01	0.01	0.01
37.97	β-Cuvebene	0.15	0.13	0.13	0.10	0.08
38.20	(E)-10-Heptadecen-8-ynoic acid methyl ester	0.01	0.01	Trace	0.01	0.01
38.45	Cedrene	0.01	0.01	Trace	0.01	Trace
38.65	Alloaromadendrene	0.17	0.17	0.16	0.15	0.14
38.78	linalyl ester	0.05	0.05	0.04	0.03	0.03
38.97	β-Bisabolene	0.05	0.05	0.05	0.04	0.04
39.25	γ-Cadinene	0.13	0.12	0.11	0.10	0.08

39.43	γ-Muurolene	0.05	0.05	0.05	0.05	0.05
39.55	(-)-Calamenene	0.02	0.02	0.02	0.02	0.02
39.64	β-Sesquiphellandrene	0.04	0.03	0.03	0.03	0.03
39.96	Juniperol	0.01	Trace	0.01	0.01	0.01
40.27	cis-α-Bisabolene	0.01	0.01	0.01	0.01	0.01
40.41	Murolan-3.9(11)-diene-10-peroxy	0.01	0.01	0.01	0.01	0.01
40.84	Caryophyllene oxide	0.04	0.04	0.04	0.04	0.05
41.37	Icosapent	0.01	0.01	0.01	0.01	0.01
41.82	Spatulenol	0.02	0.02	0.02	0.02	0.03
42.07	Caryophyllene oxide	0.42	0.39	0.42	0.41	0.45
42.55	(-)-Spathulenol	0.01	0.01	0.01	0.02	0.02
42.96	Alloaromadendrene oxide	0.02	0.02	0.02	0.02	0.02
43.33	Cubenol	0.06	0.06	0.06	0.06	0.06
44.19	Caryophyllene oxide	0.02	0.02	0.02	0.02	0.02
44.33	τ-Cadinol	0.59	0.57	0.59	0.56	0.59
44.83	τ-Muurolol	0.12	0.12	0.12	0.12	0.13
45.4	isoaromadendrene epoxide	0.07	0.07	0.08	0.07	0.08
45.92	α-Bisabolol	0.90	0.85	0.89	0.83	0.86
46.34	Carotol	0.05	0.04	0.04	0.04	0.04
46.94	aromadendrene oxide	0.01	0.01	0.01	0.01	0.02
47.99	Longipinocarvone	0.02	0.02	0.02	0.02	0.02
